# Effects of Preoperative Magnesium Sulphate on Post-Cesarean Pain, A Placebo Controlled Double Blind Study

**Published:** 2015-03

**Authors:** Seyed Mohamad Mireskandari, Khalil Pestei, Asghar Hajipour, Afshin Jafarzadeh, Shahram Samadi, Omid Nabavian

**Affiliations:** Department of Anesthesiology, Tehran University of Medical Sciences, Tehran, Iran

**Keywords:** Magnesium sulfate, Opioid, Cesarean, Pain, General anesthesia

## Abstract

**Objective:** To study the role of preoperative intravenous magnesium sulphate in decreasing post-cesarean pain and opioid requirement during first 24hrs.

**Materials and methods:** In a double blind randomized clinical trial, prior to induction of general anesthesia, fifty elective cesarean candidates were randomly assigned to one of the two groups of placebo or magnesium sulfate. After surgery visual analogue scale (VAS) and infused morphine by PCA during 24 hrs were recorded. The data were analyzed by mann-Whitney -test, analysis of variance, and student t- test.

**Results:** VAS was significantly lower among patients in the magnesium sulphate group at intervals of 1^st^, 6^th ^& 12^th^ hours after cesarean section (C/S) with the mean scales of (48.9 ± 19.6 VS 74.7 ± 18.4), (42.1 ± 0.9 VS 58.3 ± 16.5) and (25.2 ± 6.1VS 30 ± 8.1) respectively and p-value of <0.001, 0.002 and 0.05 respectively. However at 24 hrs there was no significant difference in VAS with mean VAS scales of 22.6 ± 4.5 VS 23.6 ± 4.9 and p-value of 0.49. The dose of infused Morphine during 24 hrs was significantly less in the magnesium sulphate group than the placebo group with the means of 4.36 ± 1.4 VS 7.02 ± 1.9 mg respectively (p < 0.001).

**Conclusion:** Administration of bolus 50 mg/kg magnesium sulphate prior to induction of general anesthesia may significantly decreased the morphine requirement during immediate post operative period and can be recommended as one of the modalities of post-operative pain control in the pregnant patients.

## Introduction

Central sensitization initiated by incision and tissue damage ends up in elevated intensity and longer duration of pain. Initiation of preventive measures prior to incision and their implementation through the perioperative and post-operative period, has a great impact in preventing the central sensitization, there by ameliorating the acute pain, hyperalgesia and chronic pain. In addition to the known therapeutic application, such as seizure control and pre-eclampsia, magnesium sulphate is lately known as NMDA (N-Methyl-D-aspartate) receptor antagonist in the spinal cord, which is considered important in the mechanism of central hypersensitivity .Therefore magnesium sulphate may alter this process, resulting in decreased central hypersensitivity and decreased opioid requirement (1-3).

The standard general anesthesia for cesarean section does not include opioid until after delivery of the fetus; this may result in increased possibility of hypersensitization, thereby increased post-operative acute pain and analgesia requirement (4). Considering the lately application of magnesium sulphate as preventive measures of pain in the past few years (5), it has been investigated as bolus and infusion, both prior to and during operations, results of which are variably different. However, the studies regarding its use, in the cesarean patients are very scarce. Therefore we aimed to investigate its efficacy in decreasing post-cesarean pain in patients undergoing anesthesia in whom, do not routinely receive opioids prior to incisions.

## Materials and methods

This double blind randomized clinical trial was approved by institutional ethics committee of Tehran University of medical sciences and written informed consent obtained. Fifty term pregnant patients with class I and II American Society of Anesthesiologist (ASA) scheduled for elective classic repeat cesarean section, were enrolled in the study. Reasons for exclusions were, Hypermagnesemia and any degree of heart blocks ruled out by 12-lead EKG, allergy to magnesium sulphate hypertension, diabetes mellitus, cardiovascular disease, renal disease, history of analgesic administration or intake during past 24hours, urgent-cesareans and multiple gestations. Prior to anesthesia all patients were instructed, regarding the aims of the study, administration of a standardized 0-100 mm VAS, post-operative pain control and patient controlled analgesia (PCA) apparatus.

With the help of a computer-generated list of random numbers, patients were assigned to either of the two groups of placebo or magnesium sulphate. Group assignment, preparation of drugs and their administration were performed by a nurse anesthetist who was neither involved nor interested by any means in the study. She kept grouping blind to all others including the patients themselves, until after the completion of study.

With the standard monitoring including pulse oximetry, EKG and noninvasive blood pressure (NIBP) and capnography in situ, patients in the control group received 500 ml of normal saline and patients in the intervention group received 50 mg/kg based on preconception weight of magnesium sulphate in normal saline made to 500 ml and infused within 15minutes. During which the patients were asked about sign and symptoms of hypermagnesemia. General anesthesia protocol was identical in all patients, with 4mg/kg sodium thiopental and 1.5 mg/kg succinylcholine, they were intubated and then atracurium 0.2 mg/kg was given for maintenance of muscle relaxation based on using a peripheral nerve stimulator (TOF-Watch, Organon, Dublin, Ireland). Anesthesia was continued by mixture of O_2_ and N_2_O along with 1% isoflurane. After delivery of the fetus and placenta, 1mg midazolam, 1 µg/kg fentanyl and 30 IU oxytocin (in 500 ml ringer solution) was administrated. Bispectral index (BIS) monitoring was used with A-2000 BIS monitor (Aspect Medical Systems Inc., Natick, MA, USA) to maintain BIS at 40-60. On termination of surgery, muscle relaxant was antagonized by 40µg/kg neostigmine and 20µg/kg atropine.

Hemodynamic variables were recorded prior to induction, after intubation, thirty minutes after induction and in post anesthesia care unit. APGAR scores were obtained at 1^st^ and 5^th^ minutes. During surgery blood loss was assessed by the amount of blood in the suctions and number of bloody gauzes and wipes by the nurse anesthetist. At the end of surgery a 100 mg diclofenac sodium suppository was given to all patients and then patients were extubed and transferred to post anesthesia care unit (PACU). Pain intensity was assessed by a 0-100 mm visual analogue scale at the end of 1^st^, 6^th^, 12^th^ and 24^th^ hours post-operatively at movement. Pain control was achieved by patient controlled analgesia apparatus with the setting being, 1mg bolus and 10 minutes lock out with maximum 4 hrs of 15 mg morphine. Total consumed morphine during 24 hours was recorded in both groups.

Considering the 80% of study power, for detection of 20% difference in the mean consumed morphine during 24 hours of post- cesarean operation and a pilot study results, the sample size was calculated to be 25 patients in each group. With the help of SPSS version 17.2 INC Chicago Il USA, the data were analyzed by Mann-Whitney -test, analysis of variance, and student t-test. P value ≤ 0.05 was considered significant.

## Results

Patient’s characteristic including age and weight and height were statistically similar. (Table1). VAS in the magnesium sulphate group was significantly less than control group patients at1^st^, 6^th^, and 12^th^ hours postoperatively (48.9 ± 19.6 VS 74.7 ± 18.4), (42.1 ± 0.9 VS 58.3 ± 16.5) and (25.2 ± 6.1VS 30 ± 8.1) respectively and p-value of < 0.001, 0.002 and 0.05 respectively (Table 3). Although at 24^th^ hours post operatively there was no significant difference in VAS between two groups (22.6 ± 4.5 VS 23.6 ± 4.9) and p-value of 0.49 (Table 3). The mean amount of morphine consumed during the 24 hours was significantly less in the magnesium sulphate group (4.36 ± 1.4) than the placebo group (7.2 ± 1.9) and the p-value was <0.001. There was no significant difference in surgery time. Surgical procedures in both the groups were performed in the same fashion. Hemodynamic variables including heart rate, systolic and diastolic blood pressure, between the two groups showed no significant differences at the recorded intervals (Table 2), however the same variable within each group had significant differences at various intervals compared to each other (Table 2). There was no significant difference in estimated blood loss and Apgar scores of neonates at 1^st ^and 5^th ^minutes between the two groups (Table 2). 

All patients in both the groups were extubed at intervals of 10-15 minutes following the end of surgery. There was no difference in the duration of PACU stay between the groups.

**Table 1:** Patients characteristics*

	Intervention group	Control group	p value
**Age (mean)**	**28.8 ± 4.7**	**27.6 ± 4.4**	**0.356**
**Weight (kg)**	**88 ± 7.6**	**86 ± 3.7**	**0.638**
**Height (cm)**	**161 ± 11.6**	**160 ± 9.3**	**0.812**
**Apgar Score at 1 min**	**8.1 ± 0.9**	**8.3 ± 0.7**	**0.692**
**Apgar Score at 5 min**	**9.7 ± 0.3**	**9.6 ± 0.2**	**0.711**
**Duration of surgery (min)**	**43 ± 9.8**	**48 ± 8.3**	**0.477**
**Estimated blood loss**	**480 ± 90**	**460 ± 80**	**0.155**

* Values are expressed as mean ± standard deviation

**Table 2:** Hemodynamic variables*

	Intervention group	Control group	P value
**SBP before induction **	**120.24 ± 12.8**	**120.04 ± 12.7**	**0.9**
**DBP before induction**	**79.6 ± 12.2**	**77 ± 13.4**	**0.4**
**SBP 1 min after intubation**	**133.8 ± 14.4**	**134.8 ± 13.8**	**0.7**
**DBP 1 min after intubation**	**89.3 ± 13.6**	**90 ± 12.8**	**0.8**
**SBP 30 min after intubation**	**116.9 ± 14.8**	**118.2 ± 13.7**	**0.7**
**DBP 30 min after intubation**	**75.2 ± 13.2**	**76.3 ± 13.8**	**0.7**
**SBP in recovery **	**123.5 ± 9.6**	**125.7 ± 9.5**	**0.4**
**DBP in recovery**	**80.7 ± 7**	**80.5 ± 7.2**	**0.9**
**HR before induction **	**88.4 ± 16.4**	**93.6 ± 14.2**	**0.2**
**HR 1 miniute after intubation**	**109.7 ± 14.4**	**113.4 ± 16.7**	**0.4**
**HR 30 miniutes after intubation **	**93.3 ± 12.1**	**100.2 ± 13**	**0.06**
**HR in recovery**	**88.9 ± 8.5**	**95.6 ± 17.5**	**0.1**

SBP: Systolic blood pressure; DBP: Diastolic blood pressure; HR: Heart rate

* Values are expressed as mean ± standard deviation

**Table 3:** Assessment of pain in postoperative period (Visual analogue score, 1-100 mm)*

	Intervention group	Control group	P value
**1 hour**	**48.9 ± 19.6**	**74.7±18.9**	**<0.001**
**6 hour**	**42.1 ± 5.9**	**58.3±16.5**	**0.002**
**12 hour**	**25.2 ± 6.1**	**30±8.1**	**0.05**
**24 hour**	**22.6 ± 4.5**	**23.6±4.9**	**0.49**

* Values are expressed as mean ± standard deviation

## Discussion

To the best of our knowledge and search, the effects of magnesium sulphate on post cesarean pain in patients undergoing general anesthesia have not yet been investigated. Our study showed that administration of 50 mg/kg magnesium sulphate bolus, prior to induction in cesarean section, can decrease the pain and opioid requirement during first 24 hours post operation, with no side effects in the mother and fetus. Cesarean patients undergoing general anesthesia, routinely experience painful stimuli during laryngoscopy, intubation and abdominal incision prior to fetal delivery, during which do not receive adequate analgesia due to fetal concerns. Therefore the noxious stimuli may cause central sensitization, leading to increased probability of acute and chronic post-operative pain (6). Magnesium sulphate is an intracellular cation with various physiologic functions such as enzyme activation, nerve signal conduction, and protein synthesis and vasomotor tonicity regulation. Magnesium sulphate has been used in various clinical situations including, preeclampsia, tocolysis, arrhythmias, myocardial ischemia, bronchial asthma and postoperative shivering (2, 7). Magnesium sulphate acts as an antagonist of calcium channels and noncompetitive antagonist at NMDA receptors (7-8). It appears that with this property, magnesium sulphate acts as preventive analgesic and may have a role in prevention of post-operative pain (3, 9). Many studies with various designs and methods about the effects of magnesium sulphate on post-operative pain have shown varied outcomes (10-11). In a study by Lee et al, was shown that magnesium sulphate at two doses of 30mg/kg and 45mg/kg bolus followed by 10mg/kg/hr and 15mg/kg/hr, beginning preoperatively is effective in decreasing the post cesarean pain (12). In another study by Ryu et al, effectiveness of 50mg/kg /bolus followed by 15mg/kg/hr of magnesium sulphate in curtailing the VAS, and analgesic consumption has been documented in gynecological surgery patients undergoing general anesthesia (13). On the other hand, two studies on cesarean and hysterectomy candidates did not prove its effectiveness in reducing post-operative pain (14, 15). Although the reasons of these discrepancies are not clearly known, but it could lie in the study designs, that it was investigated in patients undergoing epidural anesthesia, making the post-operative pain less pronounced. In the context of study designs and anesthesia plan, our study is similar to study by Ryu et al, with the following two differences, first we used magnesium sulphate as preoperative bolus alone, and second we administered diclofenac suppository at the end of surgery to all patients. It has been proposed that magnesium sulphate administered as a single bolus could block NMDA receptor at spinal cords, thereby decreasing the acute post-operative pain (6, 16).

The dose of magnesium sulphate administered in our study was within the usual range used in the treatment of premature labor and preeclampsia, that has been documented to be safe both in the mother and fetus (13-15).Therefore we did not measure the serum level of magnesium sulphate, and fortunately none of the patients had clinical signs of increased serum level of magnesium. All the newborns had normal APGAR scores at 1^st^ and 5^th^ minutes, with no significant difference between the two groups.

In our study, morphine requirement during 24housrs and VAS score at 1^st^, 6^th^ and12^th^ hours postoperatively was significantly less in patients who received magnesium sulphate, compared to those who received placebo. Considering that the VAS score at 24^th ^hours was similar in both groups, we state that magnesium sulphate can ameliorate the pain during first 12hours, when it is at its maximum intensity.

Taking into consideration that the magnesium sulphate is relatively a costless drug with minimal side effects and also the need to avoid the sedative effects of opioids on both mother and fetus during the immediate perioperative period, we recommend it to be one of the useful modalities in the control of post cesarean pain.

Regarding the hemodynamic effect of magnesium sulphate, many studies have shown to cause decrease in systemic blood pressure, probably due to vasodilatation, mainly arterial (17-19). However in our study, the hemodynamic variables, were similar in both the magnesium sulphate and placebo groups of patients. This may be due to the fact that we administered 500ml normal saline along with magnesium sulphate; in addition, we limited administration of benzodiazepines and opioids prior to birth. These have caused the undesired effects to be masked and not detected.

The effect of magnesium sulphate on enhancement of muscular blockade by non-depolarizing muscle relaxant has been documented in many studies (20-22). Neuromuscular monitoring is the standard monitoring, especially when magnesium sulphate has been administered perioperative. Our results showed no difference in the required neuromuscular blockade agent, between the two groups. However, we did not find any significant difference in duration of anesthesia, emergence and PACU stay, between the two groups. These results may be explained by the single bolus dose of magnesium sulphate used in our study compared to other studies that have used infusion doses.

In conclusion, we showed that administration of 50 mg/kg intravenous bolus magnesium sulphate prior to induction of general anesthesia in elective cesarean candidates, could curtail the acute post- operative pain and has sparing effects on morphine requirement during first 24hrs.
